# Interferon Regulatory Factor 5 in the Pathogenesis of Systemic Lupus Erythematosus

**DOI:** 10.1155/2012/780436

**Published:** 2012-11-01

**Authors:** Candace M. Cham, Kichul Ko, Timothy B. Niewold

**Affiliations:** ^1^Section of Rheumatology, Gwen Knapp Center for Lupus and Immunology Research, The University of Chicago, Chicago, IL 60637, USA; ^2^Division of Rheumatology and Department of Immunology, Mayo Clinic, Rochester, MN 55905, USA

## Abstract

Systemic lupus erythematosus (SLE) is an autoimmune disease characterized by multiple genetic risk factors, high levels of interferon alpha (IFN-**α**), and the production of autoantibodies against components of the cell nucleus. Interferon regulatory factor 5 (IRF5) is a transcription factor which induces the transcription of IFN-**α** and other cytokines, and genetic variants of IRF5 have been strongly linked to SLE pathogenesis. IRF5 functions downstream of Toll-like receptors and other microbial pattern-recognition receptors, and immune complexes made up of SLE-associated autoantibodies seem to function as a chronic endogenous stimulus to this pathway. In this paper, we discuss the physiologic role of IRF5 in immune defense and the ways in which *IRF5* variants may contribute to the pathogenesis of human SLE. Recent data regarding the role of *IRF5* in both serologic autoimmunity and the overproduction of IFN-**α** in human SLE are summarized. These data support a model in which SLE-risk variants of IRF5 participate in a “feed-forward” mechanism, predisposing to SLE-associated autoantibody formation, and subsequently facilitating IFN-**α** production downstream of Toll-like receptors stimulated by immune complexes composed of these autoantibodies.

## 1. Introduction

Systemic lupus erythematosus (SLE) is a complex and heterogeneous disease characterized by a strong genetic contribution and activation of a number of immune system pathways [1–3]. Recent advances in human genetics and gene expression studies have increased our understanding of the immunopathogenesis of the disorder [4]. Interferon (IFN)-*α* is a pleiotropic type I IFN with the potential to break self-tolerance by inducing dendritic cell differentiation, which can lead to the activation of autoreactive T and B cells [5, 6]. Serum IFN-*α* levels are often elevated in lupus patients [7–9] and the “IFN-*α* signature” of gene expression in peripheral blood mononuclear cells is present in more than 50% of SLE patients [10–14]. High IFN-*α* levels are associated with more severe disease and presence of particular autoantibodies [9, 14, 15]. Additionally, high levels of IFN-*α* are common in unaffected SLE family members, suggesting that IFN-*α* is a heritable risk factor [8, 16]. Moreover, some patients given recombinant human IFN-*α* for viral hepatitis C or malignancy have developed *de novo *SLE and recovered after the IFN-*α* was discontinued [17–19]. This body of evidence suggests that IFN-*α* plays a key role in etiology and pathogenesis of SLE. 

Interferon regulatory factor (IRF) 5 is a transcription factor that can induce transcription of IFN-*α* mRNA [20]. Perhaps not surprisingly, numerous genetic studies have supported an association between SLE and various single-nucleotide polymorphisms (SNPs) and functional variants in the *IRF5* gene. These genetic associations have been demonstrated across multiple ancestral backgrounds, although the exact molecular mechanisms by which these polymorphisms contribute to human disease pathogenesis are still unclear [21–33]. Other autoimmune diseases such as rheumatoid arthritis, Sjogren's syndrome, systemic sclerosis, multiple sclerosis, and inflammatory bowel disease have also been associated with *IRF5* polymorphisms, suggesting a role of IRF5 in common autoimmune disease pathways [34].

Like other IRF family members, IRF5 has a prototypical helix-loop-helix and a conserved tryptophan repeat in its aminoterminal DNA-binding domain. IRF5 induces gene expression by binding to promoters containing the IFN-stimulated response element (ISRE), whose consensus sequence is GAAANN [35] and AANNNGAA [36]. IRF5 has been called the “master regulator of proinflammatory cytokines” [37] because of its role in upregulating expression of IL-6, IL-12b, IL-17, IL-23, TNF-*α*, IFN-*β*-IP-10, MCP1, and RANTES [38, 39] in addition to type 1 IFN [40]. Because *IRF5* is an IFN-induced gene, its expression can potentially be enhanced via a positive feedback loop, where IFN-*α* production could lead to increased IRF5 expression and subsequently additional IFN-*α* transcription [41]. In addition, IRFs play an important role in the regulation of cell growth and apoptosis as evidenced by IRF5 playing a role in the induction of apoptosis in cancer cells [42]. While IRF5 functions in cell cycle processes and apoptosis, for the purpose of this paper we will focus on how *IRF5* relates to IFN-*α*, and how IRF5 variants may influence the pathogenesis of SLE.

## 2. IRF5 and Infection

Early and accurate detection of microbial pathogens is a critical part of the immune response against pathogens. This is accomplished through the recognition of common microbial molecules called pathogen-associated molecular patterns (PAMPs) [43]. Pattern recognition receptors (PRRs) are cell surface proteins on innate immune cells that detect these PAMPs, bind them, and subsequently set off signaling cascades to initiate the immune response. PRRs include Toll-like receptors (TLRs), C-type lectin receptors, retinoic acid-inducible gene (RIG)-I-like receptors, and nucleotide-binding oligomerization domain-(NOD-) like receptors. Many downstream targets of PRRs are members of the IRF family. Type I IFN and pro-inflammatory cytokines produced downstream of PRR ligation coordinate the recruitment of other innate and adaptive immune cells, which enable the attenuation and eventual eradication of the infection.

Studies by multiple investigators show that IRF5 in particular can be induced in response to specific viral infections such as Newcastle disease virus (NDV), vesicular stomatitis virus, and herpes simplex virus type 1 [20, 41, 44]. IRF5 expression is mainly restricted to dendritic cells, B cells, macrophages, and monocytes [39, 41], a pattern which is unique from other IRF family members. Although IRF5 expression may be constitutive, its activity must be induced via several posttranslational modifications at multiple amino acid residues (discussed below). 

## 3. IRF5 Is a Downstream Target of TLR7 and TLR9 

**Figure 1:**
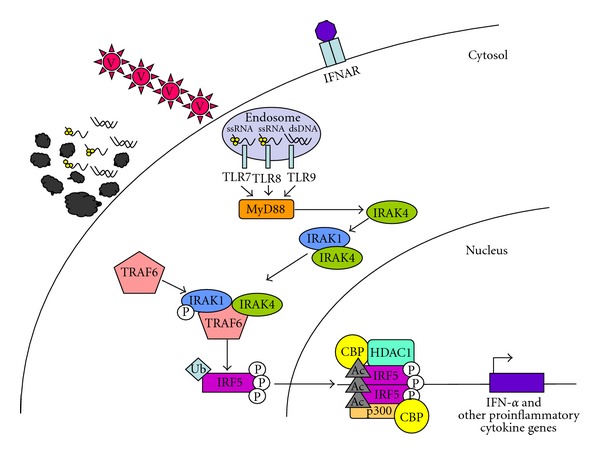
Schematic model forIRF5 activation. Cells use TLRs as sensors to detect the presence of viruses (V) via TLR7, -8, and -9. Alternatively, apoptotic debris (shown here as membrane blebs, ssRNA, and dsDNA) can also be a source of nuclear proteins and nucleic acids. Nuclear material is brought to the endosome, triggering TLR7, -8, and -9 signaling. Binding of cognate ligands to these TLRs recruits MyD88, a main signaling intermediate involved in TLR7, -8, and -9 signaling. MyD88 recruits interleukin-1 receptor associated kinase (IRAK)-4. IRAK-4 binds and phosphorylates IRAK-1, which in turn recruits Tumor necrosis factor (TNF) receptor associated factor (TRAF) 6 [46–48]. TRAF6 is an E3 ubiquitin (Ub) ligase that adds K63-Ub chains to IRF5 [49]. IRF5 is then shuttled to the nucleus and is acetylated by CBP and p300 [50]. Together, these events set the stage for the transcription of IFN-*α* and other pro-inflammatory cytokine genes.

Rather than being on the cell surface, TLR7, 8, and 9 are localized in the endosomal compartment, along with TLR3. TLR7 and TLR8 recognize single-stranded RNA viruses, while TLR9 recognizes double-stranded DNA (dsDNA) viruses or CpG motifs on bacteria. As shown in Figure 1, IRF5 is activated following engagement of TLR7 or 9, and perhaps TLR8. Of note, early studies in the characterization of TLR7 and TLR9 were performed in mutant mice [45, 46], but there is no mouse ortholog of TLR8. Therefore, less is known about the regulation and downstream signaling of TLR8, which is expressed only in humans. 

In human plasmacytoid dendritic cells (pDCs), recognition of cognate TLR7 and TLR9 ligands leads to the activation of IRF5 [51], via the signaling intermediate MyD88. As an adaptor protein that has a Toll/interleukin (IL)-1 domain, MyD88 recruits interleukin-1 receptor associated kinase (IRAK)-4. IRAK-4 binds and phosphorylates IRAK-1, which in turn recruits tumor necrosis factor (TNF) receptor associated factor (TRAF) 6 [46–48]. TRAF6 is an E3 ubiquitin (Ub) ligase that adds K63-Ub chains to IRF5 [49]. Together, these events set the stage for the translocation of IRF5 into the nucleus. 

## 4. Activation and Regulation of IRF5

Regulation of IRF5 activation is still not well understood. The C-terminal end of IRF5 has been shown to be autoinhibitory in an IFN-*α* reporter assay [41, 52, 53]. Upon stimulation, IRF5 is modified posttranslationally by phosphorylation and ubiquitination. Multiple phosphorylated residues have recently been identified (alignment positions based on IRF5 variant (v)5: T10, S158, S309, S317, S451, and S462) [54]. An additional putative phosphorylation site has been proposed at S430 on IRF5v4 (equivalent to S456 on IRF5v5) [55]. However, the importance of each phosphorylation event on IRF5 function is not clear. Chen et al. hypothesized that phosphorylation at these positions facilitated the unfolding of the auto-inhibitory structure of IRF5 monomers, promoting self-dimerization, and exposing a surface for CREB-binding protein (CBP)/p300 binding (see Figure 1 below) [55]. While there is no doubt that IRF5 is phosphorylated following stimulation through TLR7 or 9 [41], which downstream kinases and at what sites remains an area of active investigation. It is possible that pathway-specific IRF5 activation is achieved through the use of different kinases, each of which would presumably phosphorylate distinct amino acid residues. As described below, progress has been made addressing this issue in the context of RIG-I and NOD pathway regulation. 

Evidence from viral stimulation and overexpression systems has shown that RIG-I pathway kinases I*κ*B kinase (IKK)-*ε* and TANK-binding kinase (TBK) 1 can phosphorylate IRF5 [41, 44, 45, 56], but this phosphorylation is not sufficient for IRF5 nuclear translocation [56]. A recent study used mass spectrometry to identify residues S158 and S462 on IRF5v5 as targets of TBK1, a kinase involved in the RIG-I pathway [54]. These events induced IL-6 transcription, but did not transactivate IFN-*α* promoter activity [44, 56]. Studies using viral stimulation have been less clear. Barnes et al. showed that NDV induced phosphorylation of IRF5 in 2fTGH cells transfected with IRF5 [41] as well as translocation into the nucleus and transactivation of an IFN-*α* reporter construct [20]. Cheng et al. demonstrated that NDV infection did not lead to phosphorylation of IRF5 in a HEC-1B/GFP-IRF5 system [44]. This discrepancy can perhaps be explained by the differences in cell type and/or cell tropism of the viruses. Interestingly, contrary to NDV infection, Sendai virus infection in 2fTGH cells led to activation of IRF3 and IRF7, but not IRF5 [20]. Moreover, IRF5 and 7 seem to have overlapping binding partners and functions, making it difficult to distinguish the dependence of either IRF on IFN-*α* transactivation [57]. To better understand the requirement of IRF5 on IFN-*α* regulation, biochemical studies need to be done in the context of *IRF*7^−/−^ cells.

As with other IRF family members, IRF5 can form homodimers upon phosphorylation. This was demonstrated in a study in which GFP- and T7-tagged IRF5 were cotransfected with IKK*ε* into HEC-1B cells. Pull-down assays with anti-T7 antibodies showed the presence of GFP-tagged IRF5 [44]. In support of this concept, crystallographic analysis of the C-terminal fragment of IRF5(v4) S430D showed the formation of stable homodimers [55]. In addition, like IRF3, IRF5 interacts with CBP/p300 [44, 55]. Size exclusion chromatography studies have shown two molecules of IRF5 S430D binding to two molecules of CBP, forming an IRF5_2_CBP_2_ complex [55]. IRF5 can also form dimers with IRF1, IRF3, and IRF7 [41, 57]. This interaction was enhanced upon stimulation with virus. Whereas binding of IRF3 with IRF5 synergistically augmented IFN-*α* reporter activity [41], IRF5/IRF7 heterodimers blocked each other's DNA-binding domains and prevented the ability of either to bind cognate DNA sequences, resulting in the repression of IFN-*α* promoter activity [57].

In addition to phosphorylation, ubiquitylation represents another important means of regulating protein expression and activity. Two types of poly-ubiquitin (Ub) chains dictate the fate of proteins: K48-Ub and K63-Ub, where the number refers to the position of the lysine (K) residue upon which the chains of Ub are built. E3 Ub ligases are responsible for adding Ub chains to either proteins destined for degradation (K48-Ub) or for activating signal transducing proteins (K63-Ub) [58]. The E3 Ub ligase TRAF6 is activated by TLR7 and 9 signaling via MyD88 and IRAK-1. The addition of K63-Ub on IRF5 by TRAF6 is necessary for nuclear translocation and IFN-*α* transactivation. Lysines 410 and 411 are putative targets of K63-Ub since mutagenesis of these lysines to arginines abolished nuclear translocation and IFN-*α* promoter activity [48]. 

K63-Ub-IRF5 could potentially be subjected to negative regulation by deubiquitinating enzymes such as TNF-*α*-induced protein 3 (TNFAIP3, also known as A20) [59]. With regard to type 1 IFN-induced gene activity, it is unknown whether TNFAIP3 can influence TLR7 and TLR9-mediated signaling via IRF5. IRF5 activity in an IL-12p40 luciferase reporter assay system was reduced with increased expression of TNFAIP3 [54]. This system utilized receptor interactive protein kinase 2 (RIP2), a kinase involved in the NOD signaling pathway. 

Trafficking of molecules in and out of the nucleus is a tightly controlled process coordinated by importins and exportins on the nuclear membrane. These proteins recognize and bind to nuclear localization sequences (NLS) and nuclear export sequences (NES) encoded in the amino acid sequence. IRF5 has one NES (IRF5 v5 aa150-LQRMLPSLSLT-160 [44, 56] and two NLS's (IRF5 v4 aa12-PRRVRLK-18 and aa398-PREKKLI-404) [41, 55, 56, 60]. A specific inhibitor of the nuclear export protein CRM1, leptomycin B (LMB), has been used to monitor IRF5 nuclear trafficking. Treatment with LMB results in nuclear retention of IRF5 [56], indicating that IRF5 is continuously exported out of the nucleus. 

Recently, investigators have presented evidence demonstrating the regulation of transcription factor activity by acetylation/deacetylation [61]. IRF1, 2, and 7 have been shown to be acetylated by histone acetylases [62, 63]. In a study by Feng et al., IRF5 appears to be one transcription factor subject to this form of regulation as well [50]. When 2fTGH cells expressing human IRF5 and either an ISRE- or an *IFNA1*-dependent luciferase reporter construct were stimulated with virus in the presence of trichostatin A (histone deacetylase (HDAC) inhibitor), luciferase activity was ablated. Furthermore, they showed that under uninfected conditions, IRF5 forms a multicomponent complex with the corepressors HDAC1, silencing mediator of retinoic acid and thyroid hormone receptor (SMRT), and Sin3a to inhibit the luciferase reporter activity. Upon infection with NDV, IRF5 binds to histone acetylase (HAT) proteins p300, CBP, and PCAF while SMRT is exported out of the nucleus. It appears that IRF5 may be acetylated at several lysine residues since an antibody against acetylated lysine, which was used to immunoprecipitate overexpressed *IRF5* fragments, pulled out both N- and C-terminal IRF5 fragments. Taken together, IRF5 activity is highly regulated post-translationally. Multiple phosphorylation, ubiquitylation, and acetylation events must all be coordinated to induce IRF5 transactivation. 

**Figure 2:**
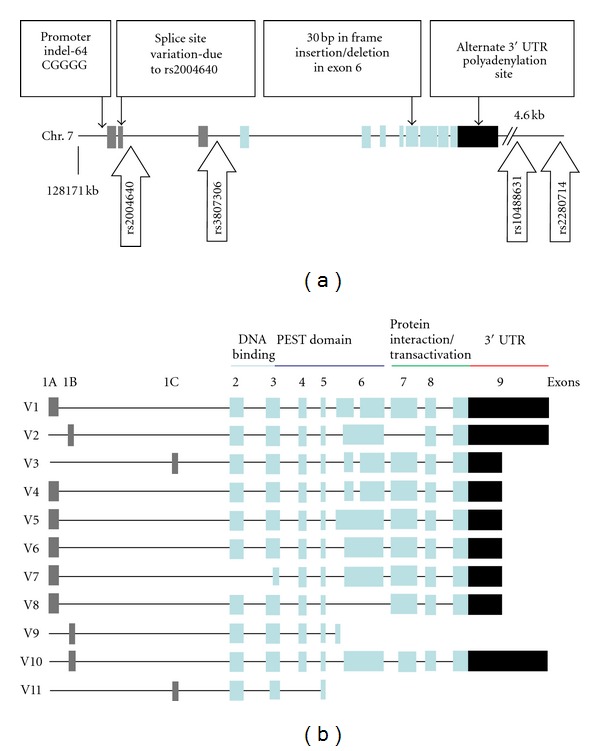
(a) *IRF5* gene marked with previously reported functional variants along with studied SNPs [64]. The first three grey boxes represent differentially spliced first exons (1A, 1B, and 1C), the next light blue boxes represent the exons 2–9, and the last black box indicates the 3′ UTR. SNPs rs2280714 and rs10488631 were used as proxies for rs10954213 in the 3′ UTR due to high LD. (b) *IRF5* mRNA isoforms [22]. There are eleven different variants. PEST, proline-, glutamic acid-, serine-, and threonine-rich.

Not only is IRF5 activation regulated by different enzymes, but also IRF5 gene expression is complex. There are up to eleven distinct isoforms of IRF5 resulting from alternative splicing [22, 60]. Four different *IRF5* transcripts result from alternative usage of the first, noncoding exon (as shown in Figure 2(b)). In the study by Mancl et al., IRF5 isoforms were differentially expressed in various purified immune cell subpopulations, though more than one isoform could be expressed in the same subpopulation [60]. For example, pDCs constitutively expressed IRF5 variants 1–4 [60]. Moreover, different IRF5 isoforms activated the IFN-*α* and IFN-*β* promoters to varying degrees, where isoform 3/4 induced the highest levels of activity [60]. In summary, many points of IRF5 regulation are possible, and greater IRF5 activity could generate an IFN-*α*-rich environment which could lead to SLE disease susceptibility.

## 5. Genetic Variants in *IRF5* Are Associated with Systemic Lupus Erythematosus

The *IRF5* locus was first implicated in SLE through a candidate gene analysis involving patients of Nordic ancestry. The SNP rs2004640 which was associated in this study introduced a new donor splice site, suggesting alternate exon 1 splicing may occur in the context of this variant [21]. A subsequent study by Graham et al. strongly replicated the association of rs2004640 with SLE in multiple independent case-control cohorts, including cases and controls from Europe, North Americans of European ancestry, and a cohort from Argentina [22]. This study also confirmed that the risk allele allowed for alternate splicing of the first exon [22]. This study described three different alternate first exons (1A, 1B, and 1C) and showed that mRNAs containing 1B could only be made when the rs2004640 risk allele was present (Figure 2(b)). The first exon is not translated, so despite this clear impact upon splicing, the functional significance of exon 1B transcripts is not clear. Even when exon 1B transcripts are produced in the setting of the splice variant, they are present at levels which are 100 times lower than those derived from other exon 1 transcripts, such as exon 1A [22, 65].

A second SNP in the 3′ region of the *IRF5* locus was associated with increased IRF5 expression [22], and an SLE-risk haplotype was described that was composed of the high expression variant of this SNP along with the alternate splice variant of rs2004640. The high expression allele was not dependent upon the splice variant in this study, suggesting that there were multiple functional elements in *IRF5*. The high expression allele was correlated with a SNP in the 3′UTR region which introduces an alternate poly-adenylation (poly-A) site and provides a potential explanation for higher *IRF5* mRNA abundance in the presence of this allele [23, 65, 66]. The SLE-risk allele of this SNP results in the production of a shorter poly-A tail, which is more stable and resistant to degradation, leading to a longer *IRF5* mRNA half-life and greater mRNA abundance (Figure 2) [23, 65]. 

## 6. Insertion/Deletion Polymorphisms in *IRF5*

In addition to the SNP variants detailed above, common insertion/deletion (indel) polymorphisms in *IRF5* have been reported, including a 30-base pair (bp) in-frame indel in exon 6, and a promoter indel [23, 28, 67]. The exon 6 insertion is present on both risk and nonrisk haplotypes. While this would suggest that it does not independently contribute to SLE-risk related to *IRF5*, the insertion is present on the risk haplotype and a cooperative role in pathogenesis cannot be ruled out. The exon 6 insertion is located in a proline-, glutamic acid-, serine-, and threonine-rich domain which can affect protein stability and function of IRF5 (Figure 2(b)) [8, 23, 67]. Moreover, a promoter indel has been described, which is 5-base long (CGGGG/−), and this insertion polymorphism in the promoter is also present on the SLE-risk haplotype. This promoter variant confers risk of SLE independently from the risk haplotype presented by Graham et al. [23, 28], as shown in Table 1. The promoter indel is in high linkage disequilibrium (LD) with the exon 1 splice site variation, and it is possible that this variant could explain the risk signal from the 5′ region of the gene (Figure 2(a)). The SLE-associated insertion creates an additional SP1 transcription factor binding site and leads to increased *IRF5* expression [28]. Whether the promoter indel or the 3′ UTR variant is more important for *IRF5* mRNA abundance is not currently understood, and SLE-associated haplotypes carry both of these polymorphisms, suggesting that both may be required to result in risk of SLE. *IRF5 *polymorphisms found to be associated with SLE in seminal candidate gene case-control studies are summarized in Table 1. Subsequent candidate gene and genome-wide association studies have strongly replicated these findings [24–27, 29–33].

## 7. Genetic Similarities and Differences by Ancestry

The risk alleles described above were initially found in European ancestry subjects, and while an association between *IRF5* and SLE has been subsequently confirmed in other ancestral backgrounds, the particular associated polymorphisms differ somewhat [24–27, 29]. For example, intron 1 SNPs (rs6953165 and rs41298401) but not exon 6 indel or 3′ UTR poly-A polymorphisms were found to be associated with SLE in Japanese population, and they were related to differential expression of several IFN pathway genes although not *IRF5* itself [26]. On the other hand, the European risk haplotype and its homozygosity appear more frequently in Mexican SLE patients compared to European patients [25], and in this ancestral background the European haplotype is a strong risk factor. In African Americans, a novel SNP rs3807306 was associated with SLE, although a functional role has not been defined [27]. We have performed follow-up work in African American and African populations which suggests that the European SLE-risk haplotype is present in African Americans due to European admixture and is associated with risk of SLE, but this haplotype was not present in African populations, and an African-derived SLE-risk haplotype was not observed in this study [23].

## 8. Autoantibodies, IFN-**α** and *IRF5* Variants

**Figure 3:**
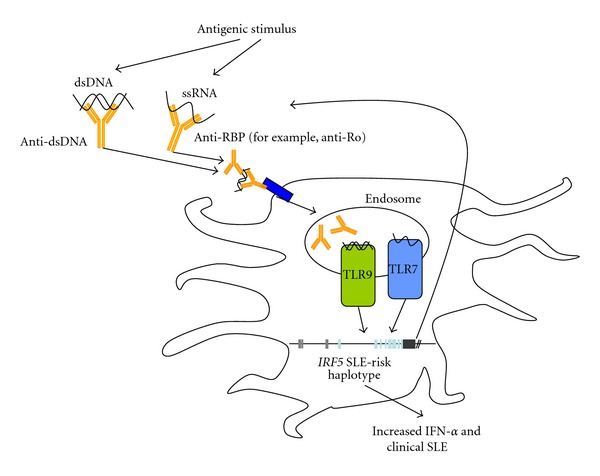
Diagram showing relationships between SLE-associated autoantibodies, *IRF5* genotype and IFN-*α* involved in the pathogenesis of SLE [64]. This suggests a “feed-forward” model in which specific auto-antibodies interact with particular *IRF5* risk variants which also predispose to the same antibody formation.

Further studies are needed to clarify how different combinations of the genetic elements of *IRF5* lead to SLE susceptibility, and what roles they play in the molecular pathogenesis of the disease. We have shown that the European risk haplotype is associated with increased serum IFN-*α* in SLE patients [68], and subsequent studies have supported this concept by showing that SLE-associated *IRF5* variants are associated with increased activation of the IFN-*α* pathway [69, 70]. However, the association between the risk haplotype and increased serum IFN-*α* in SLE patients was only observed in those patients who had anti-dsDNA or anti-RNA-binding protein (RBP) autoantibodies [68]. We expanded these findings in a study involving 1034 and 555 SLE patients with European and African ancestries, respectively [64]. The functional variants and SNPs studied are depicted in Figure 2(a). As shown in Table 2, the previously reported SLE-risk haplotype TACA [23] was associated with anti-dsDNA and anti-Ro antibodies, whereas the TATA haplotype which has previously been reported as a neutral haplotype [23] was associated with anti-dsDNA antibodies in case-case analysis. Similar patterns were detected in case-control analysis where the TACA and TATA haplotypes were associated with anti-dsDNA positive patients versus controls (Odds Ratio (OR) = 2.79, *P* = 2.9 × 10^−20^) and the TACA haplotype with anti-Ro positive patients versus controls (OR = 2.57, *P* = 1.8 × 10^−14^). The TACA haplotype is characterized by the presence of all four functional variants, the insertions in the *IRF5 *promoter and exon 6, the spice variant, and the poly-A variant, whereas the TATA haplotype has all but the exon 6 insertion [64]. The fact that these two haplotypes which differ only at the exon 6 insertion are associated with different autoantibody profiles suggests a functional relevance of the exon 6 insertion. Functional studies of the exon 6 insertion to date support a role for exon 6 variants in altering its nuclear translocation, impacting apoptosis and cytokine production [67]. Moreover, our study showed that the haplotypes associated with particular auto-antibodies resulted in increased levels of serum IFN-*α* only in the presence of that particular associated autoantibody. The above data support a pathogenic model in which these auto-antibodies chronically stimulate the endosomal TLR system, and specific *IRF5* variants in conjunction with particular autoantibodies dysregulate IFN-*α* production, resulting in increased risk of SLE (Figure 3) [64]. 

The data presented above support a “gene + autoantibody = high IFN-*α* and risk of SLE” model, and presumably the associations between *IRF5* genotype and autoantibodies may be due to this interaction. Based upon these data, we cannot rule out the possibility that *IRF5 *risk genotype could directly predispose to the formation of SLE-associated autoantibodies. In fact, *IRF5* knockouts of murine SLE models have decreased levels of SLE-associated auto-antibodies [71, 72]. This may be due to the role of IRF5 in regulating transcription of *Prdm1* which encodes Blimp-1, an essential regulator of plasma cell differentiation [73]. To answer this question in humans, we studied *IRF5* genotype in a unique cohort of anti-Ro autoantibody positive European subjects who carried a variety of diagnoses, including many who were asymptomatic and generally did not have high levels of circulating IFN-*α* [74]. We found that the *IRF5* SLE-risk haplotype was enriched even in these asymptomatic subjects with positive anti-Ro antibody, and that this enrichment was even greater (OR ~ 5) in those initially asymptomatic Ro-positive individuals who later developed SLE [75]. Taken together, these data support a “feed-forward” hypothesis in which the risk haplotype predisposes to the formation of autoantibodies, and these autoantibodies subsequently lead to increased production of IFN-*α* in conjunction with the same *IRF5* variant (Figure 3) [75].

## 9. Conclusions

In this paper, we examined how IRF5 is regulated and activated, and how its genetic variants can influence the risk of SLE by differentially activating the IFN-*α* pathway along with affecting the production of SLE-associated autoantibodies. The above data support an interesting novel model of SLE pathogenesis, in which genetic variations lead to serologic autoimmunity, subsequently creating a microenvironment which stimulates PRRs and results in high IFN-*α* [76]. 

A number of other SLE-associated genetic variants in the IFN-*α* and PRR pathways result in increased IFN-pathway activation [77–82], further supporting the concept that gain-of-function polymorphisms in the IFN-*α* and PRR pathways contribute to SLE susceptibility. While the exact initial trigger of autoimmunity in SLE remains unclear, possible antigenic sources include ultraviolet light, viruses, and demethylating drugs [83]. Recently, several studies point toward neutrophils as a factor in lupus pathogenesis [84, 85]. It has been hypothesized that chronic activation of neutrophils by immune complexes via Fc receptors induces them to release neutrophil extracellular traps (NETs) in a suicidal process called NETosis. NETs contain genomic DNA, providing a source of antigenic self-DNA. These would in turn stimulate TLRs on pDCs, putting in motion a vicious cycle of increased IFN-*α* and eventual autoimmune disease.

It is clear that IRF5 is a major pathogenic factor in human lupus, which will impact upon aspects of SLE diagnosis, prognosis, and management. Predictive models which include autoantibodies, IFN-*α* and other molecular measurements, and genetic variants may prove useful in diagnosis or prognosis. It seems unlikely that a purely genetic model will be sufficiently predictive, but the work summarized here demonstrates how other molecular phenotypes can greatly enhance the predictive capacity of genetic data. Additionally, the pathway in which IRF5 functions is currently being targeted by therapeutics directed at the endosomal TLRs and IFN-*α* [86, 87], and it is possible that *IRF5* genotype may help to define responder/nonresponder groups with respect to these therapies. The complexity demonstrated by this one disease-associated locus is staggering and suggests that we still have much work to do in understanding the genetic basis of human autoimmune disease.

## Figures and Tables

**Table 1:** Summary of genetic variants found in early seminal studies.

	Ancestry	Samples	Study type	Genetic variants	OR, *P* values	Functions
Sigurdsson et al., 2005 [21]	Swedish, Finnish	589 cases 377 controls	FB and CC association	rs2004640	OR = 1.59 *P* = 7.1 × 10^−7^	Altered exon 1 spicing

Graham et al., 2006 [22]*	Argentina, Spain, Sweden, USA	1661 cases 2508 controls	CC association	rs2004640	OR = 1.45 *P* = 4.4 × 10^−16^	Altered exon 1 splicing

		555 trio pedigrees,		Risk haplotype	OR = 1.78 *P* = 1.4 × 10^−19^	Altered exon 1 splicing, exon 6 in, short poly-A
Graham et al., 2007 [23]**	USA, UK, Sweden	2188 cases	FB and CC association	Protective haplotype 1	OR = 0.76 *P* = 5.0 × 10^−8^	Nonaltered exon 1 splicing, exon 6 in, long poly-A
		3596 controls		Protective haplotype 2	OR = 0.76 *P* = 2.8 × 10^−5^	Nonaltered exon 1 splicing, exon 6 del, short poly-A

Sigurdsson et al., 2008 [28]***	Sweden	485 cases 563 controls	CC association	CGGGG/−	OR = 1.69 *P* = 4.6 × 10^−9^	Promoter indel
				rs10488631	OR = 2.07 *P* = 9.4 × 10^−10^	Altered exon 1 splicing, exon 6 in, short poly-A

*The populations were mostly of European ancestry.

**Only the haplotype analysis is shown here. SNP rs2070197 was found to be a proxy for the risk haplotype.

***SNP rs10488631 is in high LD with rs2070197 and was used as a proxy for the risk haplotype. OR and *P* values are obtained from nonconditional analysis.

FB: family based, CC: case-control, OR: odds ratio, *P*: *P* value, poly-A: poly-adenylation, in: insertion, del: deletion, indel: insertion/deletion, LD: linkage disequilibrium.

**Table 2:** European ancestry case-case analysis showing *IRF5* haplotypes with associated functional elements and serological associations [64].

Tag SNP haplotype	Promoter indel	Splice variant	Exon 6 indel	Poly-A variant	Serologic association
(1) TACA	In	Present	In	Present	Anti-Ro: OR = 1.50, *P* = 2.0 × 10^−3^ Anti-dsDNA: OR = 1.51, *P* = 7.4 × 10^−3^
(2) TATA	In	Present	Del	Present	Anti-dsDNA: OR = 1.68, *P* = 4.9 × 10^−5^
(3) TCTA	Del	Present	In	Absent	Anti-La: OR = 3.51, *P* = 7.5 × 10^−3^
(4) GCTA	Del	Absent	Del	Present	—
(5) GCTG	Del	Absent	In	Absent	—

The haplotypes are shown as each of the four alleles in order from 5′ to 3′ (rs2004640, rs3807306, rs10488631, rs2280714).

SNP: single nucleotide polymorphism, indel: insertion/deletion, Poly-A: poly-adenylation, In: insertion, Del: deletion, OR: odds ratio, *P*: *P* value.
